# Review of Dr. C. C. F. Gay’s Article upon “Uterine Surgery,” in the March Number of the Buffalo Medical & Surgical Journal

**Published:** 1868-04

**Authors:** Frank W. Abbott


ART. III.—*Review of Dr. C. C. F. Gay's Article upon "Uterine Surgery," in the March Number of the Buffalo Medical & Surgical Journal.* By Frank W. Abbott, M. D.

When an article on any particular branch of medicine appears in a medical Journal, the natural and reasonable expectation of the reader is, that the author has either something new to communicate, drawn from his own observation and found useful in his own practice, or has studied faithfully the latest deliverances of acknowledged authorities, and has collected from them such facts and theories as are of practical or speculative interest. Moreover, when the author speaks in a tone of authority and assumption, it is but natural to infer that he is an acknowledged authority in the branch of which he treats among those whose names appear as contributors to the Journal, and is the exponent of their best ideas and practice. With these thoughts in mind, let us look for a few moments at an article on "Uterine Surgery, by C. C. F. Gay, M. D.," which appeared in the March number of this Journal.

The author proposes, as we learn from the first paragraph, to show that "our theory" of uterine disease "has been founded on a false pathology, and that such theory has led to erroneous treatment," and that the practitioner of the present day, whether engaged in general practice or devoting some attention to uterine disease as a specialty, "must lay aside old theories," "dispose of preconceived ideas," "put away old instruments to rust, and begin *de novo* to study up the true pathology and treatment of uterine disease." Surely this arraignment of the whole medical profession for ignorance and false practice must be supported by pretty strong proof to be received as true. Let us look at his statements, one by one, to see how he has sustained his position.

He commences by speaking of dislocations of the fundus uteri (the os remaining in position we suppose) as retroversions and anteversions, while those who have hitherto been regarded as authorities, call these displacements flexions, and limit the word version to those mal-positions where the whole body is displaced, constituting a very different condition. One who recognized the difference between versions and flexions would not thus confound them. But with reference to the treatment of these displacements, he says that "Simpson's sound *must* be laid aside," giving as a reason that it causes much pain and may produce pelvic cellulitis. We venture the assertion, appealing for proof to the general experience of the profession, that in the great majority of cases the introduction of the sound by skillful hands is not attended with pain, and when the uterine canal is of normal size its use is safer than the use of the probe, as it fills the canal, and is less likely to be caught in the lacunæ or folds of the mucus membrane lining the cervix or body. The pathognomonic physico-phyiognomy of the anteflected female, as detailed in the next paragraph, must have been introduced as a bit of burlesque, for, since pain in the top of the head accompanies many forms of uterine disease and other diseases, even in that part of the human family who have no uterus, it is manifestly absurd to call it pathognomonic of any particular disease.

As to the statement, that as yet nothing but failure has attended efforts to permanently restore the uterus to its normal position, the author has probably been unfortunate in his authorities, as we can point out to him, in his own city, women who have been permanently relieved by the use of the sound, together with stem and other pessaries and appropriate general treatment, and have since borne children. While we are waiting for some surgeon to gain immortal honor by discovering an operation for the radical cure of these displacements, is it not best to continue to use the measures which have often been found to fulfill the requirements of the case? While we would reprobate the use of the sound in careless or unskillful hands, we think that it should not be laid aside, for, the dislocated uterus does sometimes retain its normal position after simply being replaced by it, and not very infrequently, if after being thus replaced it is kept in position by stem pessaries, etc., and we think also that by no other means can we so easily and safely diagnose displacements of the uterus, whether idiopathic or caused by hæmatocele or pyocele, etc., or intra-uterine tumors or polypi.

The writer closes this part of his article with the satisfactory exclamation, that he has accomplished his aim of showing that many appliances, now in use for the relief of displacements of the fundus uteri, have given way to the more effectual procedure of surgical interference; but after carefully re-reading his article we fail to find that he has mentioned any surgical interference whatever, but on the contrary he says, we must lay aside the instrument which in other hands has accomplished the desired object.

The writer then turns to the subject of abrasions, congestions and ulcerations of the os uteri, proposing to treat of them practically, and not from a pathological or etiological stand-point, but what has become of his opening proposition, viz: to show that our false pathology has led to erroneous treatment? We have not found any pathology, so far, unless it may be in the case of the aforesaid pathognomonic physico-physiognomy.

Briefly stated, the assumptions of the next paragraph amount to this: Ulcerations of the os uteri may be healed in a very short time, but when they appear to be healed they are not healed, for ulcers of the os are not ulcers of the os, but only manifestations of disease within or beyond the cervix. The inconsistency of these assumptions is only equaled by the error of the last, for it is the testimony of those who have made careful *post mortem* examinations, that in ulcerations of the external os, the disease but very rarely extends beyond the cervix.

But we hasten to see what of practical good is to be learned from this apostle of progress, and the first thing is, that he has applied nitrate of silver every four or five days for three months, before effecting a cure, and the next thing is that "glycerine" or "equal parts of iodine and ol. morrhuæ" will in three days heal an "ulcerated point" caused by the application of nit. of silver. We are not yet utterly overwhelmed by the proof of our ignorance, for we have heard of applying nit. of silver to the os uteri before, and also that the "ulcerated point" caused by its application will in three days heal spontaneously.

In his paragraph on leucorrhœa, the author speaks as though it were the result simply of a diseased os or cervix or lining membrane of the uterus, and to be treated solely by local applications, utterly ignoring the general causes which may produce it, as phthisis and other lung diseases, valvular disease of the heart, etc., or more local causes, as irritation from excessive coition, polypi and fibrous tumors, retro and ante-versions and flexions, catarrhal inflammation of the vagina and diseases of the neighboring parts, as hæmorroids, vesical catarrh and calculi, etc., etc., and yet he set out with the proposition that the whole profession must learn *de novo* the true pathology of the special subject concerning which he assumes to write, without giving a single fact to sustain his assumptions.

But as it may sometimes be necessary to apply medicaments to the interior of the uterus, let us look at the instruments which he has invented to see whether they supply a vacant place in our armament. If he had attended lectures at the Medical College in his own city at any time during the past fifteen years, he might have learned to use Lallemand's straight *porte caustique* for precisely the same purposes for which his first instrument is designed, with this advantage, that the caustic may be covered until the precise point desired is reached; and to supply the place of the second he might have seen whalebone probes of any required size which by simply heating may be bent to any angle, and they have this advantage, that the cotton adheres better to them than to silver, and they are also cheaper.

With regard to the kind of speculum to be employed, the writer certainly has a right to his opinion that the cylinder and bivalve have had their day, but the assertion that Sim's or Emmet's is "the best by all odds in all cases," unsupported by a single argument, is rather sweeping. They certainly are invaluable in some operations, but American ladies, as a rule, will not submit to the presence of a competent assistant, and for ordinary purposes the cylindrical speculum, which may be used with the patient lying supine, and which brings into full view the os, without pulling it out of place with a tenaculum, is so much more convenient than the complications of the Emmet speculum, with the patient in the uncomfortable semi-prone position, that we doubt whether the profession generally are willing to exchange the one for the other, unless some better reason be given than the mere assertion that the new one is by all odds the best.

As for the cotton stem pessary, it has for a long time been the custom and teaching of some of our physicians to form a stem by twining a thread around a part of the cotton, *to rest on the perineum, or a perineal band*, to keep the ball in contact with the os uteri, and the ends of the thread are to be left long enough to be easily laid hold of, but the idea of leaving a stiff handle sticking out far enough to be seized, we are sure never entered their heads, when for this purpose a bit of tape attached to the cotton ball would do just as well. Imagine the sensation experienced in walking about for a day with such a handle protruding far anough to be grasped!

We will only call our author's attention to the concluding statement of this paragraph, which is, that by the use of his applicator, treatment has been made so effective that diseases of the os uteri, without limitation, can be cured in two or three weeks. This sounds so much like a patent medicine advertisement that we would fain believe our author spoke inadvertently.

He assumes again, in the next paragraph, that diseases appearing on the os are *only* an index and sequence of diseases within the cervix, but if we should judge of the truth of this assumption, simply by the truth of the illustration, the case would go against him, for the observations of Beaumont on Alexis St. Martin, demonstrated that the condition of the tongue is not at all an index of the pathological condition of the stomach. As a matter of practical interest we would like to inquire of our author, whether in a case of apthous stomatitis he really considers the mucous membrane of the stomach to be the seat of an exudation similar to that on the tongue, and uses remedies simply with a view to their local action on the walls of the stomach? With regard to remedies adapted to local application, chromic acid is worthy of a more extended use than it now has, and its judicious use has long been taught in Buffalo and elsewhere. It seems to attack diseased tissue more quickly than healthy, probably because its vitality is less; but if any one trusting to this should thrust a swab, wet with equal parts of this acid and water, through the os uteri, and allow the fluid which is pressed out by the pressure of the cervix around the applicator to flow down into the vagina, we fear that a vaginitis would supervene, which would be very likely to necessitate rest and the recumbent position for several days. When our author speaks of mopping out the cavity of the uterus in this heroic manner, is he ignorant of the fact, that while the application of powerful escharotics to the os and cervix uteri is rarely attended with danger, the mucous membrane lining the fundus is much more susceptible, and that their application here is likely to be followed by severe endo-metritis? Has our author ever tried the ground glass points which are used by several of his cotemporaries, by which the acid can be applied, in just the quantity and strength desired, to the particular point where it is needed?

The paragraph concerning the use of the actual cautery reads more like a pandering to common prejudice than an argument for the real good of the suffering, for the cautery is not only one of the least painful of escharotics, but it supplies a place not filled by any other. We can refer to cases of hard fibrous tumors, and soft tumors with broad bases which have been removed and healthy action established by three or four applications of the cautery, which would almost certainly have resisted the action of other escharotics for months and perhaps years, and we have also seen patients immediately after its application walk a mile or so, to their homes without inconvenience. Is this more shocking to civilization and a cause of greater disrespect to science than to apply a caustic which produces such pain that some days are required for recovery? But as a word of friendly warning, we would bid the Doctor beware how he recommends the use of the actual cautery on the brute creation lest he fall under the ban of the S. F. T. P. O. C. T. A.

This closes the practical part of the article. There only remains for us to give a brief comparison of what was proposed, with what was accomplished. It was proposed to show that our theory rests on a false pathology, which leads to erroneous practice. Not an item of commonly received pathology has been proved false, or a new one advanced unless it be the bare statement, unsupported by a single fact, that disease appearing on the os is only a manifestation of disease within the cervix and body. He speaks with disfavor of two methods of treatment, viz: the use of the sound and the actual cautery, and of the arguments against these we will speak soon. As to the instruments which he would have us lay aside to rust, he confesses that nothing which has as yet been discovered accomplishes that which is often done by the aid of Simpson's sound, and gives no reason why in ordinary cases Sim's or Emmet's speculum should be preferred to the cylinder. As for practical points, he speaks of treating leucorrhœa, without discrimination, by making applications within the cavity of the uterus with instruments of his own invention, while the use of instruments as good for this purpose, if not better, has been taught in this city for years; his use of the cotton stem pessary is simply absurd.

In regard to applications, he recommends the almost indiscriminate use of chromic acid, the discriminate use of which has long been known to his contemporaries, and speaks of iodine and sol. of the subsulphate of iron in combinations which are not new, while he condemns the actual cautery which we know from personal observation to be very useful; and this closes his teachings covering seven pages of the Journal. *Parturiunt montes nascitur ridiculus mus.*

We find not a single new fact of practical value drawn from his own experience or the writings of others, and our object in writing this review is, that such an article shall not go forth unchallenged, as an exponent of the ideas and practice of the contributors to the Journal, or the physicians of Buffalo.

